# 

**Published:** 1917-10-13

**Authors:** 


Rates of Subscription: Twelve months, 6/6; Six months, 4/-; three months, 2/-, post free to any address in the United Kingdom. Abroad, Twelve months, 8/8; Six months, 5/-; Three months, 2/6, post free. THE HOSPITAL "Index" to Volume LXI., October 1916 to March 1917, is now ready, price 2½d. per copy, post free. Address The Manager, The Hospital, "The Hospital" Building, 28/29 Southampton Street, Strand, London, W.C. 2.

### BRITISH HOSPITALS ASSOCIATION.

A conference will be held at Westminster Hospital on Friday, October 19, at 3.30 p.m., to consider (1) an increase of grants for the treatment of soldiers; (2) the proposed payment for discharged soldiers. The chair will be taken by Mr. H. Wade Deacon, chairman of the Royal Infirmary, Liverpool, and the Rev. G. B. Cronshaw, chairman of the Radcliffe Infirmary, Oxford, will open the discussion.

### HOSPITAL AND INSTITUTIONAL RESULTS.

**"Baldwin Brown" Home, Herne Bay.**—"Amid the excursions and alarums of war the home has pursued its peaceful way;" 360 ailing men, women, and children were treated at a cost of £310 19s. The average cost, including full board and the entire working expenses of every kind and sort, was 8s. 9d. per head per week.

**Royal Hospital for Sick Children, Glasgow.**—Four wards have been requisitioned by the military authorities for the use of sick and wounded officers. In the remaining wards 2,249 children were treated during 1916. The ordinary income and expenditure account shows a deficiency of £2,565. The cost per occupied cot at the country branch rose from £48 10s. 3d. in 1915 to £64 13s. in 1916.

**Warneford, Leamington, and South Warwickshire General Hospital.**—The report is abridged; lists of subscriptions and donations can be inspected at the hospital. The total income (including £7,050 legacies) was £14,086, and the expenditure £7,313. The Linen League provided 1,164 articles.

**Royal Sea-Bathing Hospital.**—"The continuance of the war, notwithstanding the burdens it imposes, does not appear to diminish but rather to increase the benevolence and beneficence of the British people." The report opens with these words, and records the work of a year in which increased expenditure has again been met by the income.

**Mothers' Hospital of the Salvation Army.**—The maternity work described in this report is carried out mainly in a building at Upper Clapton. At present there are beds for fifty cases, but the full scheme when completed will provide accommodation for 180 mothers.

**St. Peter's Hospital, Covent Garden.**—Income and expenditure nearly balanced, each being a little over £5,100. The hospital derives rents amounting to £490 from shops on the ground floor, and £2,624 from patients' payments. The beds were kept well filled during 1916, and the number of out-patients was a little higher than in the previous year.

**Hounslow Hospital**—Annual subscriptions have risen from £237 in 1914 to £289 in 1916. The total income was £1,536, and the total expenditure £1,535.

**Gordon Hospital for Rectal Diseases, Vauxhall Bridge Road.**—The annual report is almost entirely statistical in character, and does not include a report of the committee of management. An average of eighteen of the twenty-five beds were used throughout the year, and the number of out-patients was higher than in 1915. Income (including £1,765 patients' payments and donations), £2,366; expenditure, £2,365.

**Royal National Sanatorium for Consumption, Bournemouth.**—The majority of the 428 patients came from thirty-one different counties in England. The total income (including patients' payments, £872) was £7,055, and the total expenditure £6,839. The report is abbreviated.

**Children's Country Holidays Fund.**—The usual detailed list of subscribers is omitted. The total income was £17,934, and the total expenditure £16,889. One boy who went to Gloucestershire said of the country-folk: "They said such a funny langwidge that we could not understand them." Seventeen thousand two hundred and sixty-four children were given holidays—more than 1,000 in excess of the total for 1915.

**Sidcup Cottage Hospital.**—The income fell short of the expenditure by £57. The number of in-patients treated fell from 152 to 108. Patients' payments amounted to £182.

**King Edward Memorial Hospital, Ealing.**—The income, £4,076, was larger than in any previous year. Payments for civil patients amounted to £403, and for soldiers to £693. The cost of provisions for patients and staff works out at barely 8s. 6d. per week. Gifts in kind amounting in value to £270 were received. The average cost of administration was 1s. 4d. per bed per week.

**Brentwood Convalescent Home.**—The institution is for London children. The report consists of the cover and two pages, having been abbreviated in the cause of economy. The home received £398 and spent £338 in 1916, and received 160 patients who stayed an average of thirty-one days each.

### Matrons' and Sisters' Appointments.

Isolation Hospital, Chester-le-Street.—Miss Edith Hodgson has been appointed matron. She was trained at the Borough Hospital, Middlesbrough, and has been staff nurse at the Isolation Hospital, Redcar, and charge nurse at the City Hospital, Wakefield.

Bingley Cottage Hospital.—Miss Elsie Andrews has been appointed sister. She was trained at the East Suffolk Hospital, where she was later sister. She has also been sister at the Seaford Auxiliary Hospital.

Devonshire Hospital, Buxton.—Mrs. A. Murray has been appointed sister. She was trained at Noble's General Hospital, Douglas, Isle of Man, afterwards becoming theatre sister there. She has also been charge nurse at the V.A.D. Hospital, Roe Lane, Southport.

Luton, Wardown V.A.D. Hospital.—Miss P. Wood has been appointed night sister. She was trained at the Derbyshire Royal Infirmary, and has served at the Royal Bucks Hospital, Aylesbury.

Wray Hospital, Wray, Lancaster.—Miss Alice A. Haynes has been appointed sister-in-charge. She was trained at the Royal Southern Hospital, Liverpool, and has been ward sister at Monsall Hospital, Manchester, and at Murrell Hall Military Hospital, Carlisle, and temporary matron at Seaham Hall Military Hospital, Durham.

**NOTE.—Particulars of Appointments for insertion in this column will be welcomed.**

### Gifts and Bequests.

The Prince of Wales has sent £100 towards the debt on the Taunton and Somerset Hospital.

Swansea Hospital will receive about £130 as a result of the Swansea fruiterers' effort for the recent Hospital Carnival.

Mr. J. T. Blair, of Whalley Range, Manchester, left £2,000 to the Manchester Royal Infirmary, and £1,000 each to St. Mary's Hospital, Manchester, and Ancoats Hospital.

Under the will of the late Mr. G. H. Ball, of Liverpool, £2,000 was left to the Royal Southern Hospital, Liverpool, £1,000 each to the Royal Infirmary, Liverpool, the David Lewis Northern Hospital, the Victoria Central Hospital; £600 was left to the Stanley Hospital, £400 each to the Dental Hospital and the Bluecoat School, and £200 to the Samaritan Hospital for Women.

The residue, which is likely to be considerable, of the estate of Mrs. Amelia Green, of Chester Place, Regent's Park, is left in equal shares amongst fourteen charities, which include the following: The London Hospital, the London Homœopathic Hospital, King's College Hospital, St. Mary's Hospital, St. Bartholemew's Hospital, St. George's Hospital, Middlesex Hospital, Westminster Hospital, the Royal Free Hospital, the British Home for Incurables, and University College Hospital.

Mrs. Catherine Hughes, of Brook Green, W., has left the following legacies amongst others: £150 to the Cancer Hospital, and £100 each to the East London Hospital for Children, the West London Hospital, St. Thomas's Hospital, St. George's Hospital, the Hospital for Sick Children, Putney Home for Incurables, the London Hospital, the National Hospital for the Paralysed and Epileptic, and Queen Charlotte's Hospital. The residue of the estate is left to King Edward's Hospital Fund for London.

### Passing Events and Topics.

### Memorial to Dr. Potter.

On September 25 Sir Dyce Duckworth unveiled a memorial in the church of St. Elizabeth, Kensington Infirmary, to the late Dr. Potter, who for thirty-seven years held the post of medical superintendent of Kensington Infirmary, and died from blood-poisoning contracted in his duties there. The memorial represents Christ, the Great Physician, healing the sick, and is a very beautiful and inspiring conception of the miracle.

### The Ivory Cross.

The Ivory Cross, which has for its primary object the provision of artificial teeth to home army men, discharged soldiers, the Mercantile Marine, and the necessitous poor, has presented its memorandum and articles of association to the dental profession, whose interest and support were solicited at a recent meeting addressed by Major Lord Greville and Lieutenant-General Sir Francis Lloyd.

### Gift of a Sanatorium.

The Marchioness of Salisbury has given the Bedfordshire, Hertfordshire, and Huntingdonshire Joint Disablement Committee a sanatorium at Hatfield for the use of men discharged from the Army suffering from tuberculosis. There is land in the neighbourhood where the men can learn farming and recruit their health.

### L.G.B.'s Claim on Army Council.

Compensation representing more than a million pounds sterling is being claimed from the Army Council by 144 authorities, through the Local Government Board, for the use of Poor-Law institutions, mainly as hospitals for wounded soldiers.

### Infant Welfare.

The National Association for the Prevention of Infant Mortality has arranged a series of elementary lectures on Infant Care at 1 Wimpole Street, W. They began on October 1 and will continue each Monday at 5.30 p.m. up to December 17.

### Institutional Economy.

The Glasgow Corporation reports that through the installation of an economiser at the Belvedere Hospital and other improvements to the boilers, a saving has been effected in one year of 1,283 tons of coal, representing the sum of £1,039.

### Sphagnum Moss.

The Scottish Branch of the Red Cross Society is appealing for assistance in gathering sphagnum moss for dressings for wounded soldiers, 354,309 of which have been sent to hospitals at home and abroad and to clearing stations.

### Captain Watkin's Military Cross.

Captain Watkin, late assistant medical officer at the Lambeth Infirmary, has been awarded the Military Cross, and has been congratulated by the Lambeth Board of Guardians on the honour bestowed upon him.

### A Charity Removed from the Register.

In pursuance of its powers under the War Charities Act, 1916, the London County Council has removed the British-American (Overseas) Field Hospital from the Register of War Charities.

### A Condition of Exemption.

The Keighley (Yorks) Military Tribunal is making it a condition of temporary exemption that men shall do one night per week as orderlies at the hospital.

